# A GFP-strategy for efficient recombinant protein overexpression and purification in *Mycobacterium smegmatis*†
†Electronic supplementary information (ESI) available. See DOI: 10.1039/c8ra06237d


**DOI:** 10.1039/c8ra06237d

**Published:** 2018-09-25

**Authors:** Anjana Radhakrishnan, Christopher M. Furze, Mohd Syed Ahangar, Elizabeth Fullam

**Affiliations:** a School of Life Sciences, University of Warwick, Coventry, CV4 7AL, UK. Email: e.fullam@warwick.ac.uk; Tel: +44 (0)2476 574239

## Abstract

One of the major obstacles to obtaining a complete structural and functional understanding of proteins encoded by the *Mycobacterium tuberculosis* (*Mtb*) pathogen is due to significant difficulties in producing recombinant mycobacterial proteins. Recent advances that have utilised the closely related *Mycobacterium smegmatis* species as a native host have been effective. Here we have developed a method for the rapid screening of both protein production and purification strategies of mycobacterial proteins in whole *M. smegmatis* cells following green fluorescent protein (GFP) fluorescence as an indicator. We have adapted the inducible T7-promoter based pYUB1062 shuttle vector by the addition of a tobacco etch virus (TEV) cleavable C-terminal GFP enabling the target protein to be produced as a GFP-fusion with a poly-histidine tag for affinity purification. We illustrate the advantages of a fluorescent monitoring approach with the production and purification of the mycobacterial *N*-acetylglucosamine-6-phosphate deacetylase (NagA)-GFP fusion protein. The GFP system described here will accelerate the production of mycobacterial proteins that can be used to understand the molecular mechanisms of *Mtb* proteins and facilitate drug discovery efforts.

## Abstract 2

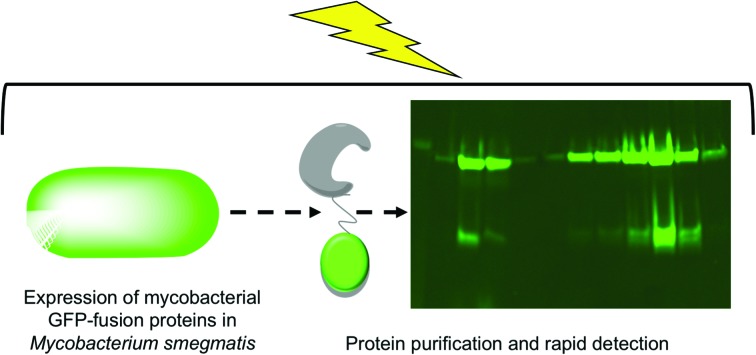
A GFP-strategy to monitor protein expression and purification in *Mycobacterium smegmatis* to overcome the obstacle of producing recombinant mycobacterial proteins.

## Introduction

Tuberculosis (TB), caused by *Mycobacterium tuberculosis* (*Mtb*), is now the leading cause of human mortality from an infectious agent. In 2016, 1.7 million deaths and 10.4 million new cases of TB were reported by the WHO.^1^ Whilst TB can be treated, the current drug-regimen is complicated and of long-duration requiring a combination of the four first-line drugs over a period of 6–9 months.^2^ Recently, there has been an increase in the emergence of numerous drug-resistant strains which further complicates the regimen, prolongs treatment and is more expensive to administer. Extensively-drug resistant (XDR-TB) strains of TB have now been reported in 123 countries and there are often no therapeutic agents to successfully treat these TB cases.^1^ Therefore, an increased knowledge of biochemical pathways employed by the *Mtb* pathogen is urgently required in order to develop new anti-tubercular agents with novel modes of action to reduce the global health threat from TB.

An important prerequisite towards obtaining a complete biochemical understanding of the *Mtb* pathogen for improved diagnostics and therapeutics is the ability to produce recombinant proteins for structural and functional studies. However, the production of high quality recombinant *Mtb* proteins in sufficient quantity has proved to be particularly challenging and has hampered progress in this area.^3^ *Escherichia coli* is one of the most commonly used bacterial host expression systems for the overexpression of recombinant proteins but often results in the production of insoluble inclusion bodies which is a major bottleneck for structural and functional studies.^4^,^5^ A number of studies have shown that the ability to produce soluble proteins from *Mtb* in *E. coli* that are correctly folded and active is particularly challenging^3^,^6^ and this has been partially attributed to the different G + C content between *E. coli* and *Mtb* which have G + C contents of 51%^7^ and of 66%^8^ respectively, although other factors may also contribute.

Given the limitations of *E. coli* as a host expression system for mycobacterial proteins a number of different systems have been explored for *Mtb* protein production. These include the use of Gram-negative *Pseudomonas putida*,^9^ Gram-positive *Streptomyces lividans*^10^ and *Rhodococcus jostii*,^11^ the yeast *Saccharomyces cerevisiae*^12^ as well as the baculovirus expression system in insect cells,^13^ although these alternative host-systems are not routinely used. It is generally considered that intrinsic difficulties in producing soluble protein can be overcome through the use of a host expression system that is more closely related to the target protein and therefore *Mycobacterium smegmatis*, which is a faster-growing mycobacterium and often used as a non-pathogenic model of *Mtb*, has been successfully utilised as a host system for the expression of a number of recombinant *Mtb* and mycobacterial proteins.^3^,^6^ To date, 55 structures of *Mtb* proteins expressed in *M. smegmatis* are now deposited in the protein data bank indicating the importance of this host-expression system for recombinant mycobacterial protein production.^14^ The advantages of using *M. smegmatis* as an expression system are numerous. The constitutive production of mycobacterial chaperones is likely to assist in the correct folding of the *Mtb* recombinant protein and is an approach that has been used successfully as a strategy in *E. coli* whereby mycobacterial chaperones are co-expressed with the target *Mtb* protein to obtain soluble proteins.^15^,^16^ Furthermore, the availability of mycobacterial specific metabolites, ligands and/or binding protein/s may lead to the production of correctly folded active protein.^17^ An example is the successful production of the *Mtb* F_420_-binding protein that could only be produced in an *M. smegmatis* host expression system and is believed to be due to the absence of the required F_420_ cofactor in *E. coli*.^18^ Furthermore, we have recently shown that the expression system can play a key role in the selection of the incorporated cofactor. Production of the mycobacterial *N*-acetylglucosamine-6-phosphate deacetylase (NagA) enzyme results in the incorporation of different metal ions depending on the host expression system with more stable, active protein produced in the *M. smegmatis* host.^19^

The vectors and strains that are currently available for the overexpression of proteins in *M. smegmatis* are not as varied or developed as the tools that are well established for *E. coli*. Recent progress in this area, however, has led to the adaption of the T7 promoter-based vector system, a commonly used system for overexpression of proteins in *E. coli*,^20^ for use in *M. smegmatis* by the introduction of the RNA polymerase from the T7 bacteriophage and the generation of the *M. smegmatis* mc^2^4517 host strain.^21^ An alternative host is the *M. smegmatis groEL1ΔC* strain that has a mutated C-terminal GroEL1 chaperone.^22^ This strain has also been developed to enable efficient purification of poly-histidine-tagged mycobacterial proteins by mutating the histidine rich region of the GroEL1 chaperone to reduce its co-purification along with the target-protein during immobilised metal affinity chromatography (IMAC) and has been used for the successful expression of mycobacterial proteins.^22^,^23^ The two main vector systems for recombinant protein expression in *M. smegmatis* are an acetamidase promoter based system and include the pSD^24^ and pMyNT^25^ series of expression vectors and the T7 promoter-based vectors that include the shuttle vectors pYUB1049 and pYUB1062,^21^ a Gateway cloning system pDESTsmg^6^ and the pYUBDUET^26^ vector for the co-expression of proteins. Although the *M. smegmatis* expression host has improved the production of recombinant mycobacterial proteins it does not always result in correctly folded proteins. Therefore, we wanted to develop a mycobacterial expression vector using GFP as an indicator to rapidly monitor protein production in *M. smegmatis*. This is an approach that has been used successfully in *E. coli* where a C-terminal GFP tag has been used as a reporter for the production of correctly folded globular and membrane proteins.^27^–^29^ A direct correlation is observed between the GFP fluorescence and the production of correctly folded protein that is fused upstream and the reported output sensitive to protein misfolding and aggregation, with no detectable fluorescence observed when the protein is expressed in inclusion bodies.

In this study we demonstrate a widely applicable, efficient protocol using GFP as a tool for monitoring the overexpression of mycobacterial proteins in *M. smegmatis*. We have adapted the inducible T7-promoter based pYUB1062 vector and incorporated a cleavable C-terminal GFP-His_6_ reporter tag to produce the target protein as a GFP-fusion. We show that protein production can be efficiently monitored by GFP fluorescence *in situ* in whole *M. smegmatis* cells. The GFP-fusion-tag does not hinder protein production and can be used to monitor purification strategies by simple procedures that include in-gel fluorescence. Taken together, our findings demonstrate that GFP is suitable for widespread use as an excellent tool for the rapid production of mycobacterial proteins in an *M. smegmatis* host.

## Materials and methods

### Materials and reagents

All chemicals and reagents were purchased from Sigma-Aldrich, unless specified. PCR and restriction enzymes were obtained from New England Biolabs.

### Bacterial strains and media

*E. coli* strain Top10 (Invitrogen) was used for the cloning of expression constructs. Transformations were selected using Luria Bertani (LB) medium containing the appropriate antibiotics. *M. smegmatis* mc^2^4517 strain (a gift from Professor W. R. Jacobs) was used for protein expression and was maintained in LB medium supplemented with 0.2% glycerol and 0.05% Tween-80 with the addition of the appropriate antibiotics.

### Construction of pYUB1062-GFP

The DNA fragment encoding the tobaccoetch virus (TEV) protease cleavage site and GFP fragment from the plasmid pWaldoD was amplified by PCR using the primers listed in Table 1. The PCR product was digested with the *HindIII* restriction enzyme and ligated into the *HindIII* site of the digested pYUB1062 vector (a gift from Professor W. R. Jacobs). The correct orientation of the TEV-GFP insert was confirmed by DNA sequencing. Subsequently, targeted site-directed mutagenesis was performed to maintain a single *HindIII* site within the multiple-cloning site using the primers listed in Table 1 and remove the second *HindIII*, incorporated during the first cloning step, with Phusion Polymerase and the PCR cycle (98 °C, 30 s; 25 cycles of 98 °C, 10 s; 60 °C, 30 s; 72 °C, 4 min; followed by 5 min at 72 °C), followed by digestion with DpnI. Plasmid sequences were verified by DNA sequencing (GATC) and removal of the additional *HindIII* site confirmed. This resulted in the formation of the resulting expression vector designated pYUB1062-GFP.

**Table 1:** Oligonucleotides used in this study. Restriction recognition sites are in italics. Codon encoding the amino acid mutation is indicated in bold type

Name	Use	Sequence (5′–3′)
GFP_pYUB1062_F	Clone GFP into pYUB1062	AAAAAA*AAGCTT*CCGAAAACCTGTACTTCCAGGGTC
GFP_pYUB1062_R	Clone GFP into pYUB1062	AAAAAA*AAGCTT*TTTGTAGAGCTCATCCATGC
pYUB_GFP_SDM_F	Mutate *Hind*III site	TGAGCTCTACAAAAAG**GGT**GCGGCCGCACTCGAGC
pYUB_GFP_SDM_R	Mutate *Hind*III site	GCTCGAGTGCGGCCGC**ACC**CTTTTTGTAGAGCTCA
NagA_pYUBGFP_F	Clone *nagA* into pYUB1062-GFP	CACCAA*CATATG*CTGCTGACCGCCGACACCGTG
NagA_pYUBGFP_R	Clone *nagA* into pYUB1062-GFP	TATAAA*AAGCTT*CCACCGTGTGCGCCGCGCCG

### Cloning of *nagA*

The full-length *N*-acetylglucosamine-6-phosphate deacetylase (*nagA*) gene from *Mycobacterium smegmatis* was amplified by polymerase chain reaction from the *MSnagA_pYUB1062* vector.^19^ The primer sequences used are listed in Table 1. The PCR products were digested with *NdeI* and *HindIII* and ligated into the pYUB1062-GFP vector digested with the same restriction enzymes, resulting in the construct nagA_pYUB1062-GFP. Plasmid sequences were verified by DNA sequencing (GATC) and used for protein expression.

### Growth curves

Growth of *M. smegmatis* in the presence/absence of pYUB1062, pYUB1062-GFP and *nagA_*pYUB1062-GFP were carried out in a 96-well microtiter plate (Falcon: black with clear bottom). The optical density at 600 nm (OD_600_) and the GFP fluorescence at *λ*_ex_ 485 nm *λ*_em_ 535 nm were monitored simultaneously every 20 min for 100 hours at 37 °C (orbital shaking 430 rpm, Tecan Infinite F200, gain 35). Cultures were grown in LB medium supplemented with 0.05% Tween-80, 0.2% glycerol with addition of the appropriate antibiotics. Induction of protein was at OD_600_ of 0.6 by the addition of acetamide (0–0.4%). All experiments were carried out in triplicate. The curves were fitted to the data points using the Lowess fit in GraphPad Prism V7.

### Protein expression of GFP and NagA-GFP in *Mycobacterium smegmatis*

*M. smegmatis* mc^2^4517 electrocompetent cells were transformed with either the pYUB1062-GFP or the nagA_pYUB1062-GFP construct and grown at 37 °C to an OD_600_ of 0.6 in LB medium supplemented with 0.05% Tween-80, 0.2% glycerol, 25 μg mL^–1^ kanamycin and 100 μg mL^–1^ hygromycin. Protein production was induced with 0.2% acetamide and the cultures were grown at 37 °C for an additional 20 hours with shaking (180 rpm). Prior to harvesting the cells, 1 mL of the culture was removed, centrifuged (10 min, 4 °C), the pellet resuspended in 100 μL PBS and the fluorescence monitored at *λ*_ex_ 485 nm *λ*_em_ 535 nm (Tecan Infinite F200, gain 35). The cells were then centrifuged (5000*g*, 30 min 4 °C) and the pellets frozen at –80 °C until further use.

### Protein purification

The cells were resuspended in lysis buffer (20 mM Tris, 300 mM NaCl, 10% glycerol pH 8.0 (buffer A)) supplemented with 0.1% Triton-X 100, Complete Protease Inhibitor Cocktail (Pierce), 5 mM MgCl_2_, 2 mg DNase and 20 mg lysozyme. The resuspended pellet was incubated at 37 °C for 10 min and the pellet passed through a Cell Disruptor at 25 kpsi at 4 °C (Constant Systems). Following centrifugation (27 000*g*, 40 min, 4 °C) the supernatant was filtered (0.45 μm pore size) before loading onto a pre-equilibrated immobilised metal affinity chromatography (IMAC) resin.

### GFP protein purification

The GFP protein was purified by loading onto a pre-equilibrated HisPur Ni^2+^-affinity resin (Thermo Scientific). The column was washed with buffer A (5 column volumes) and the recombinant GFP protein was eluted from the Ni^2+^-resin with increasing concentrations of imidazole. Fractions containing the purified GFP protein as determined by SDS-PAGE were dialysed at 4 °C for 12 hours against buffer A and the concentration of the protein determined.

### NagA protein purification

The lysate containing the NagA-GFP-His_6_ protein (∼69 kDa) was loaded onto a pre-equilibrated HisPur Co^2+^-affinity resin (Thermo Scientific). The column was washed with buffer A (5 column volumes) and the recombinant NagA-GFP-His_6_ protein was eluted from the Co^2+^-resin with increasing concentrations of imidazole. Fractions containing the NagA-GFP-His_6_ protein were dialysed at 4 °C for 12 hours against 20 mM Tris–HCl, 100 mM NaCl, 10% glycerol pH 8.0 (buffer B) at 4 °C for 12 hours and applied to a HiTrap Q-column (1 mL, GE Healthcare Life Sciences) pre-equilibrated with buffer B and eluted with NaCl (0.1–1 M). Fractions containing NagA-GFP-His_6_ were pooled and purified further using size exclusion chromatography. Gel filtration experiments were carried out on a Superdex 200 16/60 column (GE Healthcare) using 20 mM Bis-Tris, 300 mM NaCl, 10% glycerol pH 8.0 (buffer C). To remove the C-terminal GFP-His_6_-tag, fractions containing purified NagA-GFP-His_6_, as determined by SDS-PAGE, were pooled and incubated for 16 hours at 4 °C in the presence of the histidine-tagged super TEV protease (1 : 30 ratio) and loaded onto a pre-equilibrated Ni^2+^-affinity resin. The column was washed with buffer C (5 column volumes) and the cleaved NagA protein (∼40 kDa) was eluted from the Ni^2+^ resin. The NagA protein was pooled, 0.03% DDM and 1 mM DTT were added and the protein concentrated to 5–10 mg mL^–1^ (Vivaspin 2, GE Healthcare) and stored at –80 °C.

### In-gel fluorescence readings

Samples for SDS-PAGE were mixed in a 1 : 1 ratio with loading buffer (100 mM Tris, pH 6.8, 4% w/v SDS, 0.2% w/v bromophenol blue, 10% v/v β-mercaptoethanol and 20% v/v glycerol) and samples (20 μL) loaded directly onto the gel (Any kD™ Mini-PROTEAN TGX Precast protein gels, Bio-Rad) without heating. Following SDS-PAGE the in-gel fluorescence was imaged immediately using *λ*_ex_ 460 nm and the detection filter 510DF10 GFP, with an exposure time of 0.25 s (ImageQuant LAS 4000). The exposure time was selected to ensure that the brightest bands were not saturated. The gels were then stained with InstantBlue™ Coomassie Protein stain.

### Circular dichroism (CD) analysis

Purified proteins (0.3 mg mL^–1^) were dialysed in 20 mM Tris, 100 mM NaCl, 10% glycerol pH 8.0 buffer, transferred into a 1 mm path length quartz cuvette and analysed on Jasco J-810 DC spectrometer from 198–260 nm. Spectra were acquired in triplicate and averaged after subtraction of the buffer background.

### NagA assay

The activity of both NagA and NagA-GFP-His_6_ enzymes was measured at 37 °C in an end point assay by following the production of the fluorescent product formed with fluorescamine and primary amines at *λ*_ex_ 340 nm *λ*_em_ 460 nm. The reaction was carried out in a 96-well microtiter plate in 20 mM Bis–Tris, 300 mM NaCl, 10% glycerol pH 7.0 in a total reaction volume of 50 μL. The reaction was initiated by the addition of *N*-acetylglucosamine-6-phosphate (GlcNAc6P) (Carbosynth) and terminated by the addition of 50 μL 0.4 M borate buffer pH 10, 40 μL 5 mM fluorescamine (Carbosynth) and 50 μL DMF and the production of fluorescence was monitored (Tecan Infinite M200, constant gain). The production of free amine was quantified with a glucosamine standard. The kinetic parameters *K*_m_ and *V*_max_ were determined by fitting the velocity *versus* substrate concentration to the Michaelis-Menten equation using non-linear regression analysis (GraphPad Prism, V7). All measurements were performed in triplicate.

## Results and discussion

### Construction of the *M. smegmatis* pYUB1062-GFP expression vector

**Fig. 1:**
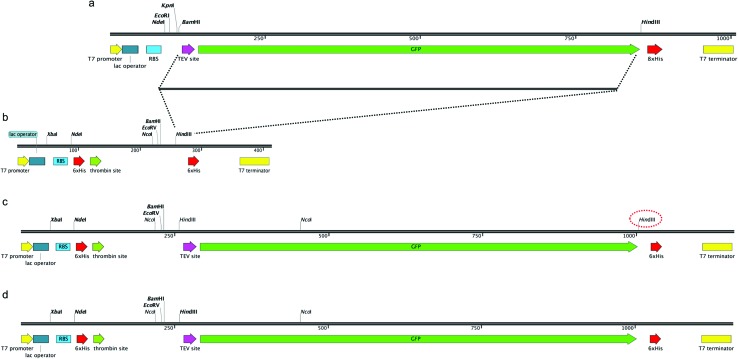
Construction of pYUB1062-GFP vector. (a) Schematic of the pWaldoD vector. (b) Schematic of the pYUB1062 vector. (c) Insertion of the TEV-DNA fragment into the pYUB1062 vector. The second *HindIII* site that is introduced is highlighted in red dashed lines. (d) The final arrangement of the pYUB1062-GFP vector.

In order to construct a fluorescent indicator system to monitor recombinant protein expression in *M. smegmatis* mc^2^4517 we selected the pYUB1062 shuttle vector,^21^ which can replicate in both *E. coli* and *M. smegmatis*, for incorporation of a C-terminal fused GFP-His_6_-tag to the target protein. The pYUB1062 vector contains a T7 promoter for inducible protein expression with either acetamide or IPTG, and a multiple-cloning site introduced from the pET30a plasmid and allows for either an N- and/or C-terminal His_6_-tag/s for purification depending on the selected cloning strategy (Fig. 1). The pWaldo vector, which contains a C-terminal GFP-His_8_ tag that can be cleaved by the tobacco etch virus (TEV) protease (Fig. 1(a)), has been used extensively to monitor the production of both cytosolic and membrane proteins in *E. coli*.^27^–^29^ In order to convert the pYUB1062 vector (Fig. 1(b)) we amplified the TEV-GFP DNA fragment from the pWaldo vector and inserted this fragment into the *HindIII* site of the pYUB1062 vector that following translation, is in-frame with the translational start-site (Fig. 1(c)) and encodes for a C-terminal His_6_ affinity-tag. The introduction of this fragment resulted in the generation of an additional *HindIII* site into the backbone of the vector (Fig. 1(c)), which was subsequently removed by using targeted site-directed mutagenesis. The resultant plasmid is shown in Fig. 1(d) and is designated pYUB1062-GFP.

### Production and purification of GFP from *M. smegmatis* mc^2^4517

**Fig. 2:**
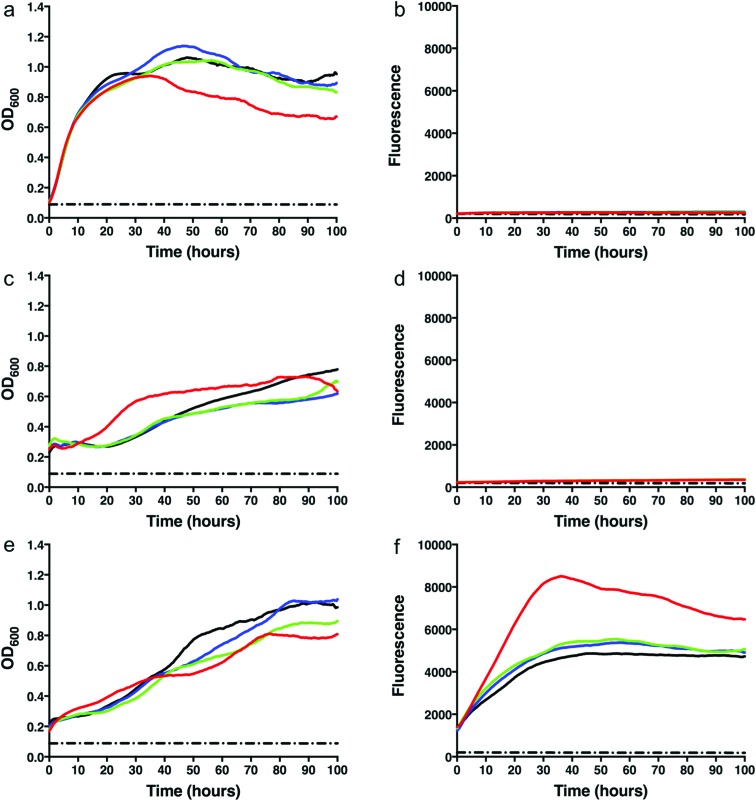
Growth rates and green fluorescent protein (GFP) fluorescence of *M. smegmatis* mc^2^4517 strains. (a) Growth curve of *M. smegmatis* mc^2^4517. (b) GFP fluorescence of *M. smegmatis* mc^2^4517. (c) Growth curve of *M. smegmatis* mc^2^4517 transformed with pYUB1062. (d) GFP fluorescence of *M. smegmatis* mc^2^4517 transformed with pYUB1062. (e) Growth curve of *M. smegmatis* mc^2^4517 transformed with pYUB1062-GFP. (f) GFP fluorescence of *M. smegmatis* mc^2^4517 transformed with pYUB1062-GFP. Concentrations of acetamide induction: red – 0%, green – 0.1%, blue – 0.2%, black – 0.4%. The dashed black line represents a media only control.

To test whether we were able to express and monitor the production of GFP *in situ* we first overexpressed GFP from the pYUB1062-GFP vector in *M. smegmatis* mc^2^4517. *M. smegmatis* mc^2^4517 strains, in the presence or absence of pYUB1062 or the newly constructed pYUB1062-GFP, were grown to an OD_600_ of 0.6 in LB expression media followed by induction with acetamide (0–0.4%). The growth rate and the fluorescence output following induction were measured simultaneously in whole cells (Fig. 2). The induction time point is defined as *t* = 0. Compared to the non-induced control, the addition of acetamide did not influence the growth of the different *M. smegmatis* strains over the 100 hours time period for all concentrations of acetamide tested (Fig. 2). Importantly, we were able to monitor the production of GFP in the pYUB1062-GFP *M. smegmatis* mc^2^4517 expression strain *in situ* (Fig. 2(f)). The GFP fluorescence increased continually until it reached a maximum level (corresponding to 13 μg GFP, 100 μL culture), as detected by fluorescence analysis, at approximately 20–30 hours after induction (Fig. 2(e) and (f)). The high level of recombinant GFP expression was not detrimental to the growth of *M. smegmatis* which is important in the development of a mycobacterial reporter recombinant protein expression system (Fig. 2(e)). We did, however, observe that GFP was expressed at the *t* = 0 induction time point and that the highest level of GFP produced were in the absence of acetamide (Fig. 2(f)), indicating that the promoter is not tightly regulated and that background expression occurs. In *E. coli* the T7/*lac* promoter is well known to result in leaky expression due to the negative control of the *lac* promoter.^4^,^30^

**Fig. 3:**
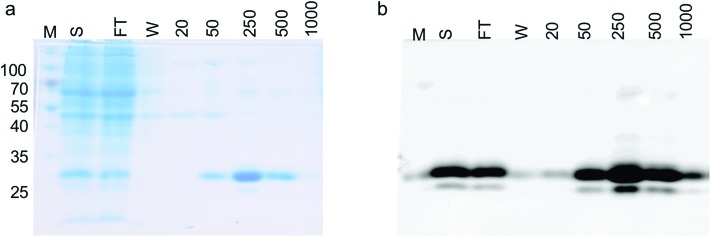
Expression and purification of GFP in *M. smegmatis* mc^2^4517. (a) SDS-PAGE analysis of the overexpression and purification of GFP-His_6_. (b) In-gel fluorescence of the overexpression and purification of GFP-His_6_. M: molecular weight markers in kDa, S: soluble lysate, FT: column flow-through, W: buffer wash-step, numbers 20–1000 refer to the imidazole concentration in the elution buffer (units mM).

To demonstrate that the GFP protein produced from this expression system can be purified and the purification steps monitored, we produced GFP in 1 L culture volume. We used the conditions optimised from our test conditions and grew *M. smegmatis* mc^2^4517 transformed with pYUB1062-GFP to an OD_600_ of 0.6 and then induced the culture with 0.2% acetamide for 20 hours. At this final 20 hour time point we measured the GFP fluorescence in whole cells which corresponded to the production of 100 mg of GFP. The *M. smegmatis* cell pellets were then lysed by cell disruption and the His_6_-tagged GFP protein purified by Ni^2+^-affinity-chromatography (IMAC) and the eluted fractions were monitored by in-gel fluorescence and Coomassie blue staining (Fig. 3). Following purification, we isolated ∼50 mg of purified GFP protein, indicating that there is a good correlation between the GFP that is detected in the whole *M. smegmatis* cells and the amount of protein purified. It is likely that incomplete cell lysis and loss of protein during the purification steps resulted in the slightly lower yield of purified GFP protein than expected. It was of interest to note that the yield of purified GFP obtained from the *M. smegmatis* expression host is comparable to the yields of GFP that we have obtained previously from *E. coli*. This indicates that the GFP-reporter for protein overexpression in an *M. smegmatis* expression system is robust and comparable to the fluorescent based systems that are routinely used for protein over-expression in *E. coli*^28^,^29^ and *S. cerevisiae* hosts.^31^

### Production of NagA-GFP-His_6_ in *M. smegmatis* mc^2^4517

**Fig. 4:**
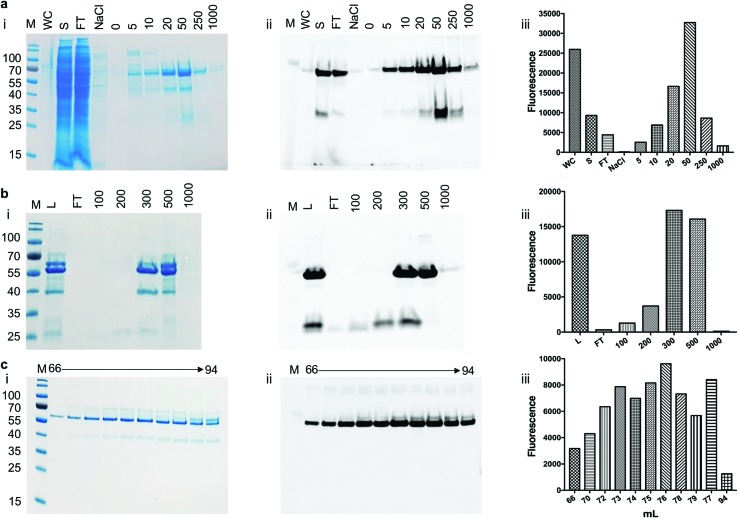
Purification of NagA-GFP-His_6_ expressed in *M. smegmatis* mc^2^4517. (a) Elution of NagA-GFP-His_6_ protein from a Co^2+^ column. M: molecular weight markers in kDa, WC: cell lysate, S: soluble lysate, FT: column flow through, NaCl: 1 M NaCl wash, numbers 0 to 1000 refer to the imidazole concentration in the buffer (units mM). (b) QHP anion chromatography of NagA-GFP-His_6_ following the Co^2+^ purification step. L: protein loaded onto the column after the Co^2+^ purification step, FT: column flow through, numbers 100–1000 indicate the NaCl concentrations in the elution buffer (units of mM). (c) Size exclusion chromatography of NagA-GFP-His_6_. (i) SDS-PAGE stained with Coomassie blue, (ii) corresponding in-gel fluorescence analysis and (iii) GFP fluorescence readings following the purification steps of NagA-GFP-His_6_.

To assess the use of the pYUB1062-GFP reporter strain for the production of mycobacterial proteins for structural and functional studies we chose to produce the mycobacterial *N*-acetylglucosamine-6-phosphate (NagA) enzyme as a GFP-fusion. We have recently produced NagA as His_6_-tag fused protein in *M. smegmatis* mc^2^4517 as soluble protein^19^ and therefore wanted to determine whether the GFP-fusion impacted on the expression yield and/or activity of this protein. To determine the optimal expression conditions for the NagA-GFP-His_6_ fusion we measured the OD_600_ and the GFP fluorescence for 24 hours in microtiter plates following induction at an OD_600_ of 0.6 with acetamide (0–0.4%) and observed that the fluorescent signal continued to increase indicating that the NagA-GFP-His_6_ protein continued to be produced over this time period (ESI, Fig. S1†). It was interesting to note that the results from this primary screen indicated that the optimal conditions to produce the NagA-GFP-His_6_ protein were identical to those used previously to produce His_6_-tagged NagA in 1 L culture volume.^19^ Following cell lysis, the NagA-GFP-His_6_ protein was purified by Co^2+^-affinity, anion exchange and size-exclusion chromatography (Fig. 4). The eluted fractions from each purification step were monitored by analysis of the fluorescence output using a plate-reader and by in-gel fluorescence following SDS-PAGE. The addition of the C-terminal GFP-tag to the NagA protein enabled the purification strategy to be optimised and monitored more rapidly compared to our previous studies with NagA as a His_6_ fusion.^19^ The quality of the protein can be assessed through a comparison of the intensity of the NagA-GFP-His_6_ fusion compared to GFP alone and has been used routinely to monitor the integrity of membrane proteins expressed as GFP proteins in *E. coli*.^27^–^29^,^31^–^34^ Our in-gel fluorescence results for the whole cell lysate indicated a prominent signal at ∼70 kDa with only low levels of GFP detected at ∼27 kDa, indicating that the NagA-GFP-His_6_ fusion protein was intact and minimal degradation had occurred (Fig. 4(a)). The size-exclusion profile indicated that the NagA-GFP-His_6_ fusion protein forms a dimer in solution (ESI, Fig. S2†) which was also observed for the NagA-His_6_ fusion^19^ and is characteristic to NagA proteins from other bacterial species.^35^,^36^ This is important and indicates that the oligomeric complex is not altered by the addition of the C-terminal GFP-tag which has also been found for proteins expressed as GFP fusions in both *E. coli*^28^,^32^–^34^ and *S. cerevisiae* systems.^31^ To obtain NagA, the purified NagA-GFP-His_6_ fusion was digested with TEV-His_6_ protease. The digest went to completion and NagA was obtained by removal of the digested GFP-His_6_-tag and the TEV-His_6_ protease by Ni^2+^-chromatography (ESI, Fig. S3†). Correct folding of the NagA-GFP-His_6_ fusion and NagA following cleavage of the GFP-His_6_-tag was confirmed by circular-dichroism (ESI, Fig. S4†). The amount of purified NagA protein that we produced following expression as a GFP fusion (∼1 mg L^–1^ culture) was comparable to that obtained as a His_6_ fusion^19^ (∼2 mg L^–1^ culture) indicating, importantly, that the GFP reporter was not detrimental to the production and purification of the NagA protein.

### Activity of NagA-GFP-His_6_

**Fig. 5:**
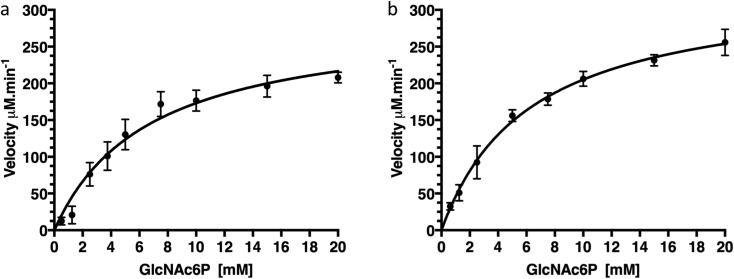
Kinetics of NagA-GFP-His_6_ before and after cleavage of the GFP-tag. (a) Kinetics of NagA-GFP-His_6_, (b) kinetics of NagA after cleavage of the GFP-His_6_-tag. Each assay was carried out in triplicate and expressed as ± standard deviation.

To confirm that production of protein fused with a C-terminal GFP reporter tag is not detrimental to activity of the protein we examined the catalytic deacetylation of *N*-acetylglucosamine-6-phosphate (GlcNAc6P) substrate by both the NagA-GFP-His_6_ fusion and the cleaved NagA enzymes using a fluorescence assay.^19^ The measurements indicated that both NagA-GFP-His_6_ and NagA were able to deacetylate GlcNAc6P with *K*_m_ values of 6.7 mM and 6.2 mM and *k*_cat_ values of 96.4 s^–1^ and 111 s^–1^ respectively (Fig. 5 and Table 2). Importantly, the kinetic values that were obtained are comparable to the values previously obtained for the NagA-His_6_ fused protein also expressed in *M. smegmatis*.^19^ This indicates that a C-terminal GFP fusion is not detrimental to the correct folding or catalytic activity of recombinant proteins expressed in *M. smegmatis*.

**Table 2:** Steady-state kinetic parameters of NagA

Enzyme	Fusion-tag	GFP-tag	*K*_m_ (mM)	*k*_cat_ (s^–1^)	*k*_cat_/*K*_m_ × 10^3^ M^– 1^s^–1^	Reference
NagA	His_6_	Not cleaved	3.2 ± 0.4	91.1 ± 5.5	28.8 ± 3.8	19
NagA	GFP-His_6_	Not cleaved	6.7 ± 1.2	96.4 ± 7.3	14.4 ± 2.8	This study
NagA	GFP-His_6_	Cleaved	6.2 ± 0.6	111.0 ± 4.0	17.9 ± 1.8	This study

## Conclusions

In this study we have developed a new method for the efficient screening and purification of mycobacterial proteins using GFP and *M. smegmatis* as a host expression system. Here we have identified that GFP can be used as a fluorescence indicator to monitor recombinant protein expression *in situ* in whole *M. smegmatis* cells and that it is possible to undertake preliminary screens in microtiter plate format for the rapid optimisation of overexpression conditions. The GFP-reporter tag is not detrimental to the growth of *M. smegmatis* and enables the purification of mycobacterial proteins to be rapidly monitored from the GFP fluorescence detection. The activity of the NagA enzyme produced as a NagA-GFP-His_6_ fusion was comparable with the NagA-His_6_ fusion indicating that the C-terminal GFP tag does not affect the biochemical function of the target protein. We hope that the availability of the pYUB1062-GFP protein expression system will facilitate the production of *Mtb*, and other mycobacterial, proteins and enable new structural and biochemical insights into this important global pathogen.

## Conflicts of interest

There are no conflicts of interest to declare.

## Abbreviations

XDR-TB: Extensively-drug resistant
IMAC: Immobilised metal affinity chromatography
*Mtb*: *Mycobacterium tuberculosis*
GlcNAc6P: *N*-Acetylglucosamine-6-phosphate
NagA: *N*-Acetylglucosamine-6-phosphate deacetylase
OD: Optical density
TEV: Tobacco etch virus
TB: Tuberculosis

## Supplementary Material

Supplementary information
