# Supplementary material for: A GFP-strategy for efficient recombinant protein overexpression and purification in Mycobacterium smegmatis
Source: RSC Adv. 2018 Sep 25;8(58):33087–95. doi: 10.1039/c8ra06237d (PMC6180428; doi:10.1039/c8ra06237d)
Supplement: RA-008-C8RA06237D-s001 [file RA-008-C8RA06237D-s001.pdf]

Electronic Supplementary Material (ESI) for RSC Advances.
This journal is © The Royal Society of Chemistry 2018

Electronic Supplementary Information

A GFP-strategy for efficient recombinant protein overexpression and purification in *Mycobacterium smegmatis*

Anjana Radhakrishnan [a], Christopher M Furze [a], Mohd Syed Ahangar [a], Elizabeth Fullam [a]*

[a] School of Life Sciences, University of Warwick, Coventry, CV4 7AL, United Kingdom. E-mail: e.fullam@warwick.ac.uk; Tel: +44 (0)2476 574239

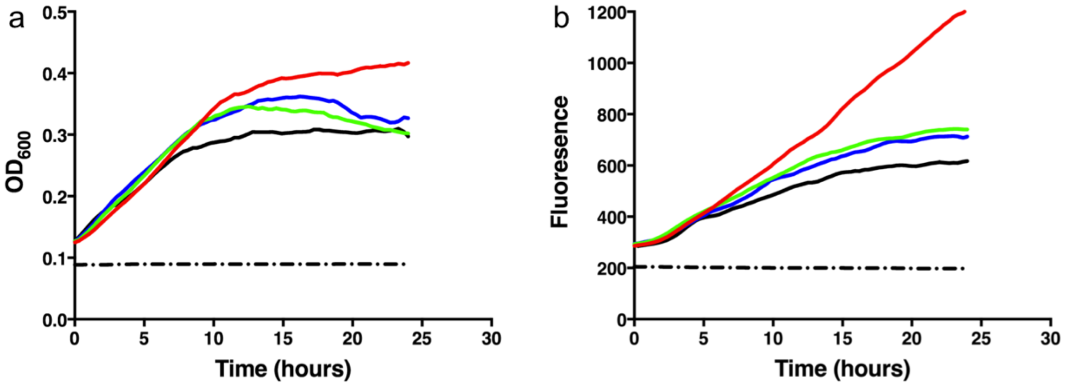

Fig. S1: Growth rates and green fluorescent protein fluorescence of NagA-GFP expressed in *M. smegmatis* mc^{2}4517 strains. (a) Growth curve of *M. smegmatis* mc^{2}4517. (b) GFP fluorescence of *M. smegmatis* mc^{2}4517. Concentrations of acetamide induction: red - 0 %, green - 0.1%, blue – 0.2%, black – 0.4 %. The dashed black line represents a media only control.

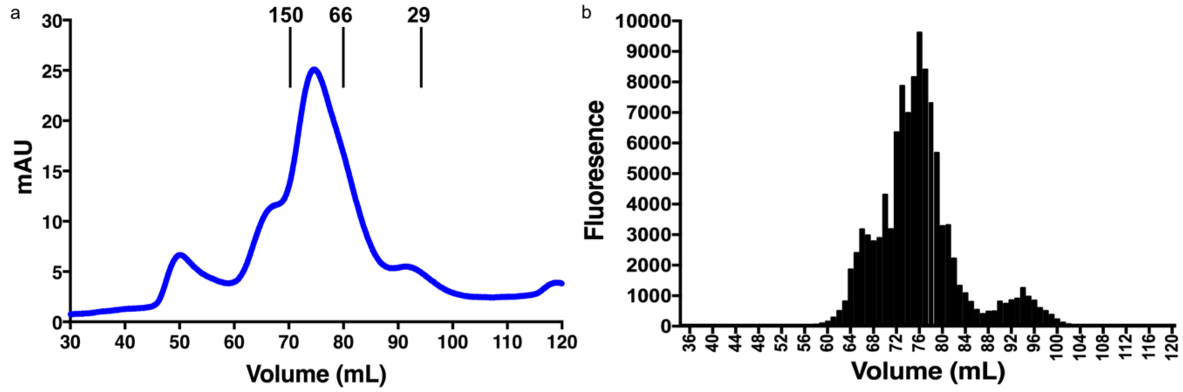

Fig S2: Size exclusion and GFP fluorescence of NagA-GFP-His_6. (a) Size exclusion chromatography of purified NagA-GFP-His_6. The sequence derived molecular weight of dimeric NagA-GFP-His_6 is 133 kDa. The elution was monitored by recording absorbance at 280 nm. (b) GFP fluorescence of the 1 mL fractions collected by size exclusion chromatography.

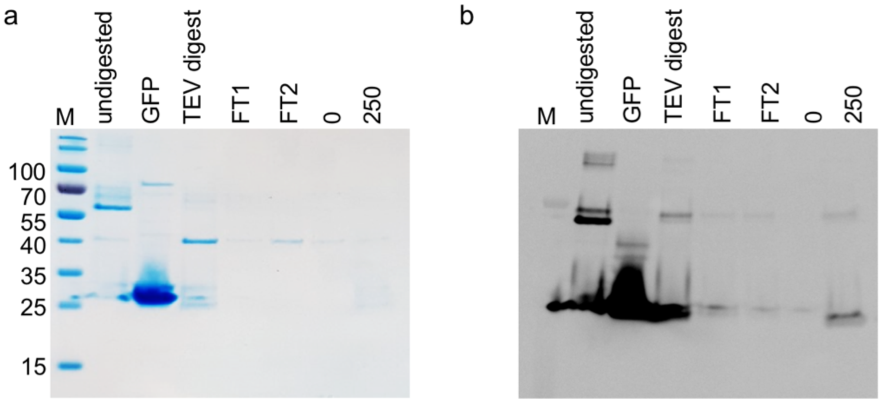

Fig S3: TEV-cleavage of NagA-GFP-His$_6$. (a) SDS-PAGE analysis of the purification of NagA-GFP-His$_6$ digested with TEV protease (b) In-gel fluorescence of NagA-GFP-His$_6$ digested with TEV protease. M: molecular weight markers in kDa, undigested: NagA-GFP-His$_6$ after size-exclusion chromatography, GFP: GFP control protein, TEV digest: NagA-GFP-His$_6$ following digest with TEV-His$_6$, FT1: flow through from column, FT2: flow through from column after reloading FT1 to the Ni^{2+}-affinity column, 0: 0mM imidazole wash-step, 250: 250mM wash-step.

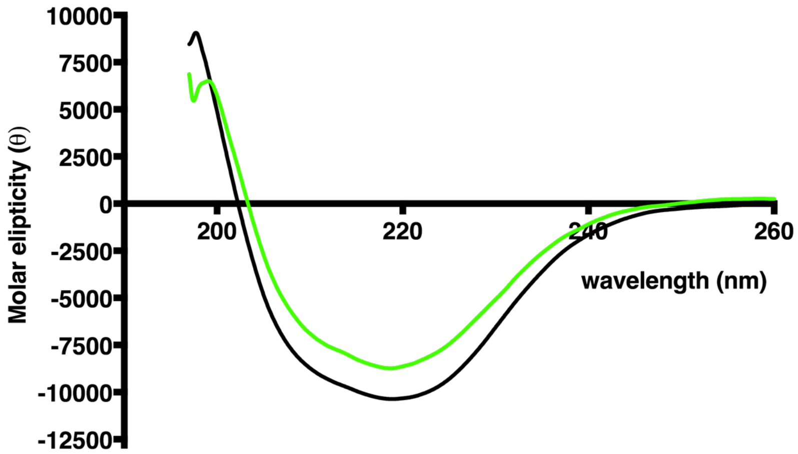

Fig S4: CD spectra of NagA-GFP-His_6 (green) and NagA after TEV-cleavage (black)
